# CDA: Combinatorial Drug Discovery Using Transcriptional Response Modules

**DOI:** 10.1371/journal.pone.0042573

**Published:** 2012-08-08

**Authors:** Ji-Hyun Lee, Dae Gyu Kim, Tae Jeong Bae, Kyoohyoung Rho, Ji-Tae Kim, Jong-Jun Lee, Yeongjun Jang, Byung Cheol Kim, Kyoung Mii Park, Sunghoon Kim

**Affiliations:** 1 Medicinal Bioconvergence Research Center, Seoul National University, Seoul, South Korea; 2 Information Center for Bio-pharmacological Network, Seoul National University, Suwon, South Korea; 3 Korean BioInformation Center (KOBIC), Korea Research Institute of Bioscience and Biotechnology (KRIBB), Yuseong-gu, Deajeon, South Korea; 4 WCU Department of Molecular Medicine and Biopharmaceutical Sciences, Seoul National University, Seoul, South Korea; University of Texas MD Anderson Cancer Center, United States of America

## Abstract

**Background:**

Anticancer therapies that target single signal transduction pathways often fail to prevent proliferation of cancer cells because of overlapping functions and cross-talk between different signaling pathways. Recent research has identified that balanced multi-component therapies might be more efficacious than highly specific single component therapies in certain cases. Ideally, synergistic combinations can provide 1) increased efficacy of the therapeutic effect 2) reduced toxicity as a result of decreased dosage providing equivalent or increased efficacy 3) the avoidance or delayed onset of drug resistance. Therefore, the interest in combinatorial drug discovery based on systems-oriented approaches has been increasing steadily in recent years.

**Methodology:**

Here we describe the development of Combinatorial Drug Assembler (CDA), a genomics and bioinformatics system, whereby using gene expression profiling, multiple signaling pathways are targeted for combinatorial drug discovery. CDA performs expression pattern matching of signaling pathway components to compare genes expressed in an input cell line (or patient sample data), with expression patterns in cell lines treated with different small molecules. Then it detects best pattern matching combinatorial drug pairs across the input gene set-related signaling pathways to detect where gene expression patterns overlap and those predicted drug pairs could likely be applied as combination therapy. We carried out *in vitro* validations on non-small cell lung cancer cells and triple-negative breast cancer (TNBC) cells. We found two combinatorial drug pairs that showed synergistic effect on lung cancer cells. Furthermore, we also observed that halofantrine and vinblastine were synergistic on TNBC cells.

**Conclusions:**

CDA provides a new way for rational drug combination. Together with phExplorer, CDA also provides functional insights into combinatorial drugs. CDA is freely available at http://cda.i-pharm.org.

## Introduction

Advances in *in vitro* test systems have shifted drug research from animal studies to target-oriented research [1]. Combining this process with genomic research, agents specifically targeting unique proteins related to specific disease have been found. Amongst these successful stories of targeted agents is the BCR-ABL kinase inhibitor imatinib (Gleevec; Novartis), which is using for the treatment of chronic myelogenous leukemia (CML). However, in such cases, drug resistance arises possibly owing to the diversity of mutations of the gene encoding BCR-ABL as well as other pathways on parallel signalling pathways [2]. Despite successes such as these, many other drug candidates targeting disease-associated gene products have been found to be inefficient or to cause severe side effects. So the limitations of the single protein targeted agent paradigm have come to surface.

Living systems rely on complex signaling pathways to maintain their performance in the face of various perturbations [3]. This complexity appears to pose a barrier for anticancer therapies targeting single signalling pathways. Cancer cells possess compensatory mechanisms to overcome perturbations where they occur at one signalling axis and so therapies targeting only one pathway can fail in clinical trials due to lack of efficacy, or be overcome by mutations at an important receptor [4]. Recent research has identified that in some cases, balanced multi-component therapies might be better than highly specific single component therapies [5]–[7]. These drug combinations are pharmaco-dynamically synergistic, additive or antagonistic as their effects are greater than, equal to, or less than the summed effects of individual drugs, respectively [8]. These models have garnered interest in the possibility of effective combinatorial drug discovery based on systems-oriented approaches [9]–[13].

Geva-Zatorsky et al. found that protein responses to combinations of drugs were described accurately by a linear superposition (weighted sum) of their responses to each drug alone [14]. With this in mind, we designed a system for multiple signaling pathways targeting combinatorial drug discovery using gene expression profile. We assumed that if there are two different drugs which regulate two different disease-associated pathways individually, combination of them might be effective unless they affect to each other in unanticipated ways. Based on this model, expression pattern matching methods should be a valuable to quantify the degree of functional similarity among genetic perturbation, disease, and drugs. However, despite current data bases of mRNA expression profiles, which contain thousands of data points, many of which are available to the public, the number of combinatorial drug discovery approaches based on expression profiles is less than might be expected.

Here we introduce the CDA, for predicting combinatorial drug candidates that target multiple signaling pathways. CDA contains 6,100 expression profiles representing 1,309 molecules which were imported from Connectivity Map [15]. When a user submits “up probe sets” and “down probe sets”, CDA starts hyper-geometric tests for signaling pathway gene set enrichment analysis. Next signaling pathway expression pattern analysis and drug set pattern analysis are performed to measure expression pattern similarity between input signatures and 6,100 expression profiles. These analyses focus on the signaling pathways which are selected in the gene set enrichment analysis (the previous step). CDA then generates lists of single drugs and combinatorial drugs showing similar expression patterns. If user input signatures are disease-related significant probe sets, high negative scoring drugs can be considered candidate drugs for treating individuals whose diseased tissues show opposite gene expression aberrations in signalling pathways as the input cell line.

We present the results in two different formats: a table view of scores and experimental details, and a network view to visualize relationships between signaling pathway entities and known drugs, proteins and diseases. phExplorer, a graphical data visualization software program, allows users to browse the complex relationships in an interactive and dynamic manner, providing clues to how chemicals work synergistically on certain signaling pathways. To validate the technique, we performed two *in vitro* combinatorial drug discovery studies, on non-small cell lung cancer cells and triple-negative breast cancer (TNBC) cells, and succeeded in each case to find combinatorial drug pairs that exerted synergistic effects in cell culture.

## Results

### Drug Combination Suggestion through Transcription Response Module Analysis

**Figure 1:**
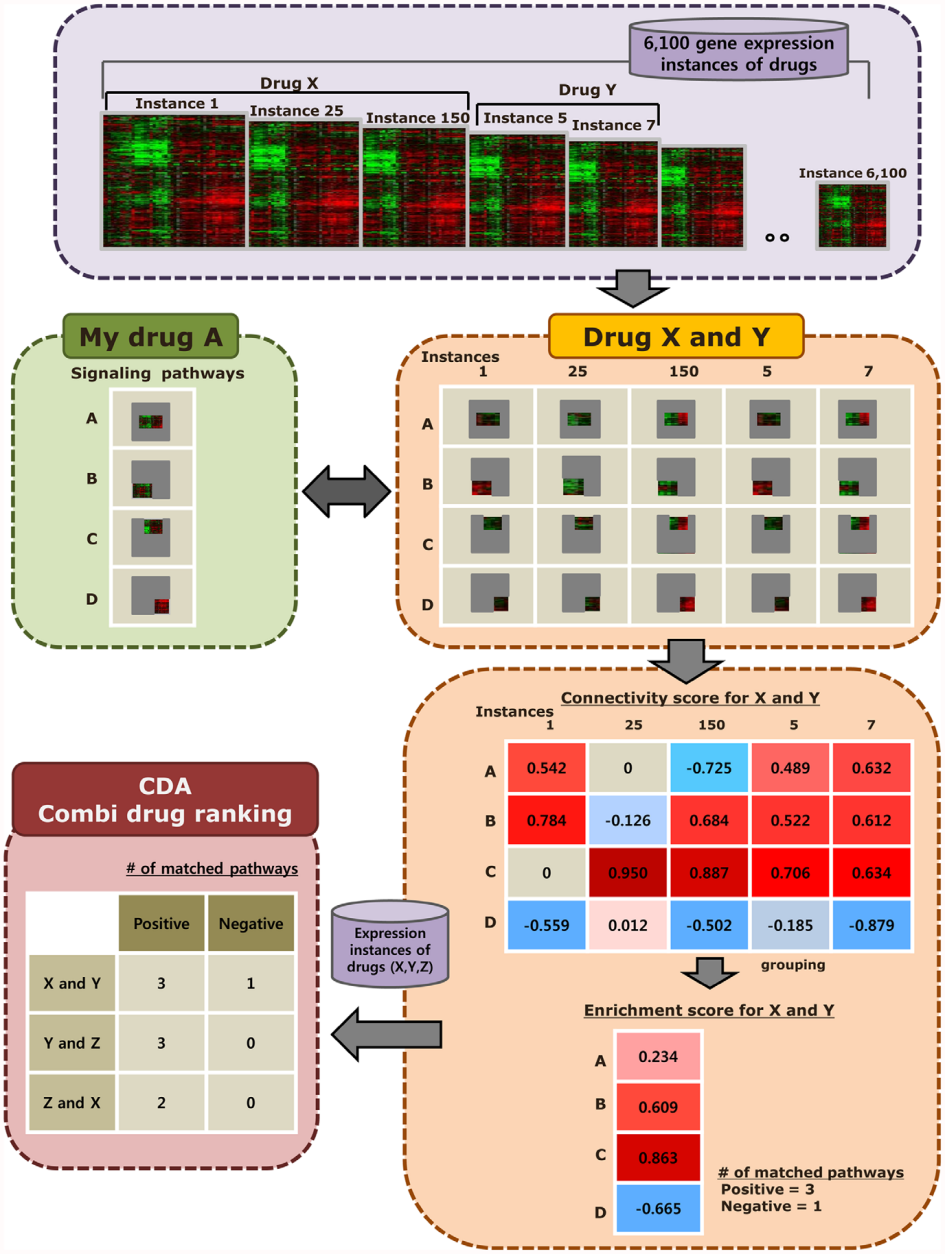
Analysis pipeline of CDA. Combinatorial drug analysis process. In drug set pattern analysis step (the bottom right box), combinatorial drug analysis process treats profiles of two different molecules as a group to measure the synergistic effects of them.

CDA uses gene expression data in cellular models to pinpoint combinatorial drug pairs that can regulate multiple signaling pathways that potentially synergize to cause disease states, or which through alternate pathways compensate to reduce the efficacy of a drug targeting only one pathway. The combinatorial drug possibility is predicted by gene expression pattern comparison within the selected disease-related signaling pathways. The possibility is scored using Kolmogorov-Smirnov statistics. CDA is composed of four steps; 1) Preparing input signatures and gene set enrichment analysis of signaling pathways 2) Pathway expression pattern analysis 3) Drug set pattern analysis 4) Counting of the number of pathways which show positive/negative correlations with input signatures for drug ranking (See methods for more details and Figure 1). To validate the technique, we performed one *in silico* single drug discovery study and two *in vitro* combinatorial drug discovery studies. In an *in silico* validation for single drug analysis, CDA successfully identified a molecule having similar function (Case one). As the discovered combinatorial drug pairs were mostly novel, we carried out *in vitro* validations on non-small cell lung cancer cells and triple-negative breast cancer (TNBC) cells (Case two and three).

### Case One: Molecules Function as Estrogen Antagonist

Elevated blood levels of estrogen is associated with an increased risk of breast cancer [16]. Gene expression signatures in breast cancer cells treated with Letrozole (fifty eight untreated tumors and fifty eight letrozole-treated tumors, GDS3116) were used to search the molecules function as estrogen antagonist [17]. Letrozole inhibits, aromatase, an enzyme that participates in estrogen biosynthesis. By inhibiting estrogen synthesis, letrozole slows the proliferations of breast cancer cells. Table 1 shows that cells treated with fulvestrant share a very similar expression pattern to those treated with letrozole. Fulvestrant is an estrogen receptor antagonist with no agonist effects. Fulvestrant not only down-regulates transcriptional activities of estrogen receptor but also induce its degradation. Fulvestrant was approved by the FDA for the treatment of postmenopausal women with hormone receptor-positive metastatic breast cancer [18]. The gene expression signatures of cells treated with fulvestrant in 6 different signaling pathways resembled those of letrozole. Not surprisingly, they show similar patterns of gene expression on the plasma membrane estrogen receptor signaling pathway as well as on LPA receptor mediated events pathway and stabilization, expansion of the E-cadherin adherens junction pathway, and Reelin signaling pathway.

**Table 1:** Top 10 molecules showing similar expression patterns of transcriptional response modules to letrozole.

	FOXM1 transcription factor network	IGF1 pathway	IL4-mediated signaling events	LPA receptor mediated events	Plasma membrane estrogen receptor signaling	Reelin signaling pathway	Stabilization and expansion of the E-cadherin adherens junction
Fulvestrant		O	O	O	O	O	O
Trichostatin A	O	O	O		O	O	
Irinotecan	O	O		O	O		O
Ag-013608					O	O	O
Tretinoin	O	O			O		
Metamizole sodium		O			O		O
Tanespimycin		O	O	O			
Vorinostat	O	O			O		
Verteporfin		O			O		
Daunorubicin		O					O

These results are illuminating in light of the connections in the literature which show these pathways are regulated by estrogen and/or involved in cancer progression. E-cadherin is a cell-cell adhesion protein, and has been shown to play a crucial role in tumor suppression [19]. A recent study by Oesterrich et al. showed that estrogen caused down-regulation of E-cadherin levels in breast cancer cells [20]. Lysophosphatidic acid (LPA; 1-acyl-glycerol 3-phosphate), which is also regulated by estrogen [21], [22] is one of the simplest natural phospholipids that mediates multiple processes including neurogenesis, angiogenesis, wound healing, and cancer progression [23], [24]. Reelin is a secreted signaling protein associated with regulation of neuronal cell positioning and migration. Its down-regulation is associated with increased migratory ability and reduced survival in breast cancer [25]. The relationship between reelin and estrogen/breast cancer is not fully understood.

Letrozole inhibits estrogen synthesis, whereas fulvestrant blocks the estrogen receptor. Although the mechanisms of those two compounds are different, the signaling cascades they affect would be expected to be similar in their down-regulation of transcriptional activity in downstream pathways. As levels of estrogen are decreased after the treatment of letrozole, signaling pathways related to E-cadherin and LPA are affected, and this perturbation in these pathways are also observed in cells treated with fulvestrant. They both regulate reelin signaling pathway to induce apoptosis in cancer cells through as yet unknown mechanisms.

### Case Two: Combinatorial Drugs that Induce Apoptosis on Tumorigenic Lung Cancer Cells

**Figure 2:**
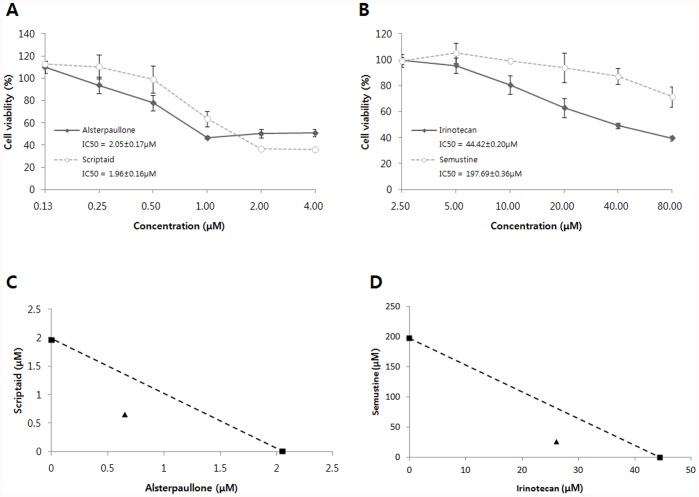
Synergistic combinatorial drug pairs on lung cancer cells. (A, B) Effects of alsterpaullone, scriptaid, irinotecan, and semustine on A549 cancer cell proliferation. IC_50_ indicates the concentration of drug that induce 50% of inhibition of cell proliferation. Error bars represent the standard deviation of six experiments. (C, D) Drug pairs were treated in 1∶1 molar ratio. The IC_50_ values of each drug are plotted on the axes, and the dashed line represents addictive effect. Triangle point represents the concentrations of the combinations resulting in 50% of proliferation inhibition. As the triangle points are positioned on the left of the dashed line, these combinatorial drug pairs are synergistic. The IC_50_ values of each drug in alsterpaullone-scriptaid and irinotecan-semustine combinations are 0.65 µM and 26.05 µM, respectively.

This case derived from a study by Landi et al. that investigated the role of cigarette smoking in lung adenocarcinoma development and survival (forty nine normal lung tissues and fifty eight lung tumor tissues, GDS3257). In our analysis, we disregarded information on smoking, disease state, and gender of the patients. In order to identify molecules that could reverse the expression pattern of lung adenocarcinoma cells, we looked for a phenotype where expression of signature genes was reversed: up-regulated genes became down-regulated, and vice versa. Signaling pathway gene set enrichment analysis of the “reversed phenotype” genes in the lung adenocarcinoma cells showed highlighted that many of the genes identified in this way are frequently associated with tumor cell growth and proliferation (Table 2). Based on this gene expression analysis, we identified, among the top 15 combinatorial drug pair candidates, two synergistic combinatorial drug pairs: alsterpaullone and scriptaid; and irinotecan and semustin. Alsterpaullone is a cyclin-dependent kinase (CDK) inhibitor that induces apoptosis [26]. Scriptaid is a class of histone deacetylase inhibitors (HDACis). HDACis are involved in cell growth, apoptosis and differentiation. Scriptaid also induces cell death in cancer cells [27], [28]. Irinotecan is an anticancer drug that binds to the DNA topoisomerase 1 complex during DNA replication, preventing the resealing of single-strand breaks [29]. Semustine also known as methyl-CCNU, is another anti-cancer drug in the class of alkylating agents [30], [31]. The alsterpaullone-scriptaid and irinotecan-semustine pairs showed meaningful, statistically significant expression pattern matching in seven, six lung adenocarcinoma-related pathways, respectively. Simultaneous and continuous exposure of A549 cells to different concentration of these two combinatorial drug pairs for 72 hours showed a synergism (Combination index (CI) <1 and Dose reduction index (DRI) >1; Table 3 and 4, Figure 2).

**Table 2:** Enriched pathway in lung adenocarcinoma.

Pathway	Pathway Category
amb2 Integrin signaling	Integrin mediated cell-cell signaling pathways Integrin mediated cell-extracellular matrix signaling pathways
Aurora A signaling	Cell cycle pathways, mitotic
Aurora B signaling	Cell cycle pathways, mitotic
BMP receptor signaling	Bone morphogenetic proteins signaling pathway
Direct p53 effectors	p53 signaling pathway
E2F transcription factor network	Transcription factor mediated signaling pathways Cell cycle pathways, mitotic Transcription pathways
Endothelins	Endothelin signaling pathway
FGF signaling pathway	Fibroblast growth factor signaling pathway
FOXM1 transcription factor network	Forkhead signaling pathways

**Table 3:** CI values for the drug combinations at 25%, 50%, 75% levels of inhibition of A549 cell proliferation.

CI Values	25%	50%	75%
Alsterpaullone + Scriptaid	0.887	0.647	0.483
Irinotecan + Semustine	0.816	0.718	0.636

**Table 4:** DRI values for the drug combinations at 25%, 50%, 75% levels of inhibition of A549 cell proliferation.

DRI Values	25%	50%	75%
Alsterpaullone + Scriptaid			
Alsterpaullone	2.013	3.162	4.968
Scriptaid	2.565	3.020	3.554
Irinotecan + Semustine			
Irinotecan	1.452	1.705	2.002
Semustine	7.841	7.589	7.345

### Case Three: Combinatorial Drugs that Induce Apoptosis on Triple-negative Breast Cancer Cells

Breast cancer is the most common form of cancer in women. Human epidermal growth factor receptor 2 (HER2), also known as receptor tyrosine-protein kinase ERBB2, belongs to the epidermal growth factor receptor (EGFR) family, and it is one of the most important oncogenes in invasive breast cancer. Based on the importance of HER2 amplification on breast cancer, the HER2-targeting monoclonal antibody trastuzumab was developed [32]. Additionally, aberrant EGFR signaling is a major characteristic of a human cancer including breast cancer. Several anti-EGFR agents are currently undergoing clinical testing in breast cancer patients clinically [33]. However, triple negative breast cancer (TNBC) is a type of breast cancers that does not express the genes for estrogen receptor (ER), progesterone receptor (PR) or human epidermal growth factor receptor 2 (HER2). For that reason, novel effective therapeutic agents are needed for TNBC patients [34]. Combined treatment of general breast cancer cells with drugs that target EGFR and HER2 results in a synergistic antitumor effect [35], [36]. That means that targeting EGFR family signaling pathway is a good strategy for breast cancer treatment.

**Figure 3:**
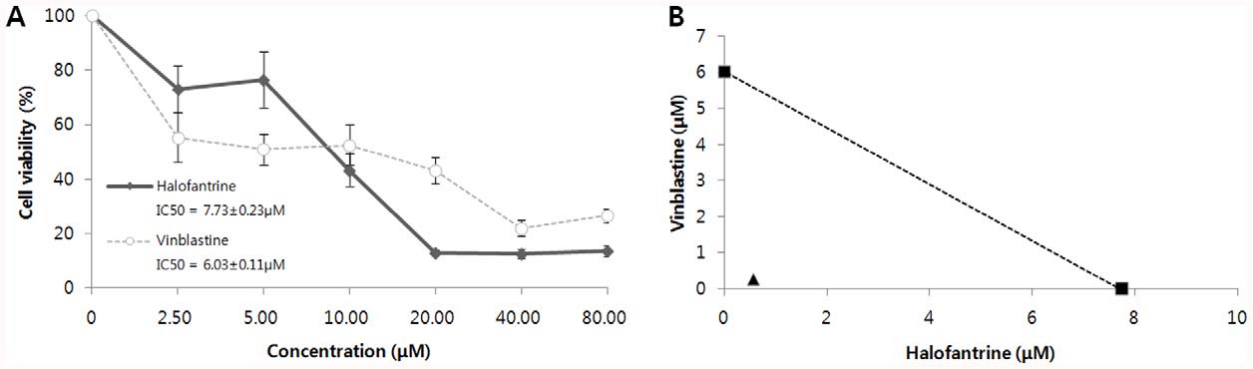
In vitro validation of halofantrine and vinblastine alone and in combination in a triple-negative breast cancer cell line. (A) Effects of halofantrine and vinblastine on MDA-MB-231 TNBC cell proliferation. IC_50_ indicates the concentration of drug that induce 50% of inhibition of cell proliferation. (B) Halofantrine and vinblastine combination was treated in 2∶1 molar ratio. Halofantrine and vinblastine combination shows a strong synergistic effect. The IC_50_ values of each drug in halofantrine-vinblastine combinations are 0.55 µM and 0.27 µM, respectively. The combination shows a strong synergistic effect (CI value is 0.12, and DRI values for halofantrine and vinblastine are 14.17 and 22.09, respectively).

**Figure 4:**
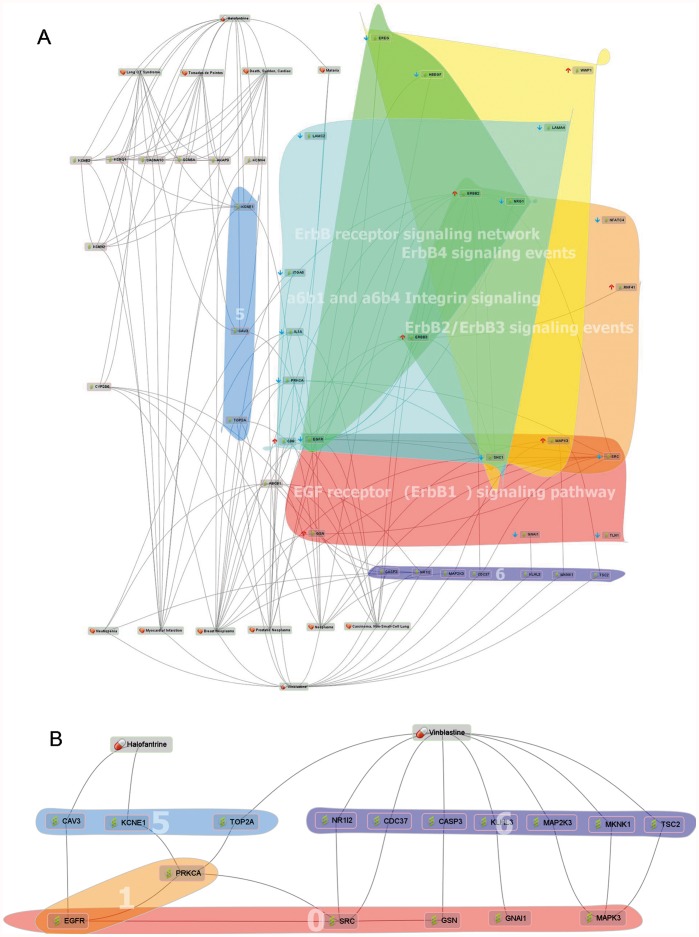
Network map of halofantrine and vinblastine on triple-negative breast cancer using phExplorer. (A) It seems that halofantrine and vinblastine could affect on five different signaling pathways in TNBC. Group 5: Halofantrine- or vinblatine-related proteins which are also related with proteins of A6B1 and A6B4 Integrin signaling pathway. Group 6: Proteins which are related with vinblasitne as well as proteins of EGFR family signaling pathways (such as ERBB1 signaling pathway, ERBB2/ERBB3 signaling events, ERBB4 signaling events, ERBB receptor signaling network). (B) We hypnotized that halofantrine and vinblastine are synergistic because they complementary regulate integrin and EGFR signaling pathways. Group 0: A part of EGFR family signaling pathways. Group 1: A part of A6B1 and A6B4 Integrin signaling pathway.

To discover a synergistic combinatorial drug pair for TNBC patients, we focused on FDA approved drugs. We obtained gene expression signatures from TNBC cell lines (five normal breast cancer cell lines and five triple-negative breast cancer cell lines, GSE6569), and we selected halofantrine - vinblastine pair as a candidate pair (Figure 3). The CDA analysis indicated that the pair has opposite expression patterns compared with TNBC signatures in five different signaling pathways, including four of the EGFR family signaling pathways and one integrin pathway (Figure 4). Aberrant activation of the EGFR family is implicated in a number of cancers and it is already the target of several antineoplastic agents [37]. A6b1- and a6b4- mediated integrin signaling is involved in apoptosis, tumour cell invasions, and cell migration.

Halofantrine is an anti-malarial agent with an unknown mode of action. Although it has cardiotoxic potential, it is safe when carefully administered [38]. Vinblastine is a microtubule-targeted anticancer drug that induces mitotic block and apoptosis by suppressing microtubule dynamics at lower concentration, and reducing microtubule polymer mass at higher concentration [39]. As shown in Figure 4B, halofantrine and vinblastine are indirectly related to EGFR family signaling pathways. Furthermore, both are also related to an integrin signaling pathway. Based on this information, we hypothesized that halofantrine and vinblastine are synergistic because they simultaneously affect the EGFR and integrin signaling pathways. Furthermore, sensitivity of HER2-positive breast cancer cells resistant to anti-HER2 therapies are related to antiapoptotic proteins MCL1 and Survivin [40]. And these two proteins commonly have protein-protein interactions with CASP3, a vinblastine-related protein [41], [42]. Based on this, we hypothesized that vinblastin could be a good TNBC drug candidate. Using the steps described for all three cases, CDA users will be able to put forward testable hypotheses by combining signaling pathway expression information with known drug-protein-disease information from phExplorer.

## Discussion

Since the number of new drug has not kept pace with the enormous increase in pharma R&D spending, drug discovery researchers have become more creative in finding new uses for existing drugs [43]. Analyzing large data sets such as gene expression [15], chemical similarity [44], side-effect similarity [45], disease-drug network [46], and phenotypic disease network [47] has been applied for drug repositioning. Exploration of drug off-targets using chemical-protein interactome can also provide alternative strategy [48]. However drugs with single targets frequently show limited efficacies and drug resistance at the some point. To overcome these problems, systems-oriented drug design is now moving to multicomponent therapies and multi-targeted drugs, based on the idea that targeting drugs to act on multiple signaling pathways will maximize therapeutic efficacy [49]. With this in mind, we have designed a system for multiple signaling pathways targeting combinatorial drug discovery using gene expression profile. There are three groups of pharmacodynamically synergistic combinations; 1) anti-counteractive action group 2) complementary action group 3) facilitating action group. There are a variety of mechanism of actions represented by these combinations, arising from drug interactions with the same or different targets of the same or different pathways, and from modulations of crosstalk pathways and network robustness [8].

The robustness of CDA does not depend heavily on the particular bioinformatics method employed for signature extraction, thus providing a flexible analysis platform that can be adopted by a variety of users with different software tools for handling gene expression analysis. Although genome-wide expression analysis has become a routine tool in genomic research, extracting biologically meaningful information remains a major challenge. Statistically significant genes can be obtained by number of different ways. Moreover, there is no standard rule to restrict the number of genes. Thus, significant gene selection is quite depending on individual researchers. Given this multiplicity of approaches, significant gene lists can be quite diverse according to extraction algorithms and research principles. This lack of standardized bioinformatics approaches brings with it a risk of insufficient information usage that can lead to inaccuracies in the final interpretation. To offset these differences, for expression analysis and interpretation, our strategy employs functionally important genes as data sets, rather than entire statistically selected gene sets. This approach was validated by an *in silico* case (Information S1). CDA provides a mechanism whereby hundreds of input signature genes will be split into signaling pathways at the first step, therefore users don’t need to themselves extract a small group of significant gene sets using number of different algorithms. Through this process, CDA successfully has identified a number of molecules having similar function (Table 1). In this study, we presented case studies whereby CDA successfully predicted synergistic combinatorial drug pairs in lung cancer and triple negative breast cancer. Together with phExplorer, CDA also provides functional insights of combinatorial drugs.

Using CDA, the number of matched pathways decides the ranking of drug candidates, however, the type of matched pathways must be considered carefully. As the interpretation of result and the final decision must be made by researchers, we tried not to restrict their choice by providing strictly ordered list based on our limited pre-knowledge.

## Materials and Methods

### Data Source

Reference molecule-treated expression data was downloaded from Connectivity Map (build 02) (http://www.broadinstitute.org/cmap/). It contains 6,100 expression profiles representing 1,309 molecules. Molecules were selectively applied to five different human cancer cell lines for short duration. Each molecule-treated expression profile was paired with a control, and each profile was represented by a non-parametric rank-ordered list of all probe sets.

Pathway gene set data was downloaded from Pathway Interaction Database (PID) on 09/03/2010 (http://pid.nci.nih.gov/). Only the NCI-Nature Curated data was used. Pathway gene set information was extracted, consisting of 166 pathways comprising 2,297 genes. These genes were annotated to Affymetrix GeneChip Human Genome U133 Array Set HG-U133A probe set. The final form of pathway data consists of 166 signaling pathways and 3,726 probe sets.

Furthermore, nine public databases, EntrezGene interaction [50], MINT [51], DIP [52], CTD [53], TTD [54], ChemBank [55], PharmGKB [56], OMIM (http://www.ncbi.nlm.nih.gov/omim/), and GAD [57] were integrated to visualise enrich drug-protein-disease network map. For data integration in a unified format, we adopted PubChem CID for drugs, GeneID for proteins, and MeSH descriptor for diseases. The integrated database is called PharmDB, and it is available at http://pharmdb.org/.

### Input Signatures

Three different GDS/GSE data files were downloaded for each case study. All of them were used Affymetrix Human Genome U133A Array.

Case 1: GDS3116 - Letrozole effect on breast cancer.

Fifty eight untreated tumors vs. fifty eight letrozole-treated tumors

Case 2: GDS3257 - Lung adenocarcinoma.

Forty nine normal lung tissues vs. fifty eight lung tumor tissues

Case 3: GSE6569 - Triple-negative breast cancer cell lines.

Five normal breast cancer cell lines: BT474, SKBR3, HCC-1419, HCC-1954, MCF7

Triple-negative breast cancer cell lines: BT20, BT549, HCC-1806, MDA-MB-231, MDA-MB-468

The expression data were normalized using RMA from the BioConductor Affy package. Then these data were analyzed using a method called empirical Bayes in limma. To extract statistically differentially expressed genes, 2-fold change and p-value <0.05 were set as default. The signatures were represented by two probe sets, “up probe sets” and “down probe sets”. With given input signatures, hyper geometric tests were performed for signaling pathway gene set enrichment analysis. Signaling pathways with p-value <0.01 were selected as it was believed that input signature genes were enriched in these pathways.

### Enrichment Analysis

Signaling pathway expression pattern analysis and drug set pattern analysis were performed based on the Kolmogorov-Smirnov statistics. To determine whether the distribution of input gene sets/or drug sets was significant, 10,000 times permutations were carried out by generating random ranking matrices. The sets with p-value <0.01 were indicated as enriched.

### Signaling Pathway Expression Pattern Analysis

6,100 molecule-treated expression profiles were rank ordered using gene set enrichment analysis for each selected pathway. As mentioned above, there were two types of input set, “up probe sets” and “down probe sets”. The expression pattern similarity is calculated for both sets. The procedure is as follows:

1 Calculate Kolmogorov-Smirnov score for both “up probe sets” and “down probe sets”

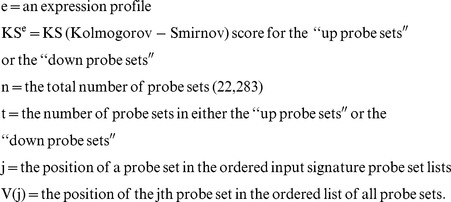


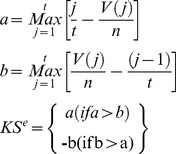




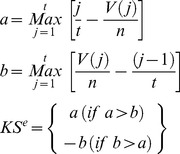

2 Calculate the Enrichment Score (ES) for each profile

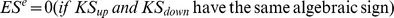
Otherwise, across all profiles,



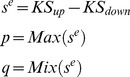
The ES for these profiles are:



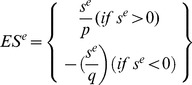

3 Rank the profiles in descending order of ES^e^

### Drug Set Pattern Analysis

Molecules were applied to different cell lines with various doses, and the ES of each molecule was calculated using the distribution of the molecule-treated profiles, using the same method as used in calculating the KS score in signaling pathway expression pattern comparison. For the case of combinatorial drug analysis, signatures of two different molecules were treated as a group. The rationale is as follows: we assume two molecules, “A” and “B” show highly similar expression pattern with the expression of signaling pathway “SP1” and “SP2”, respectively. The purpose of combinatorial drug is matching up two molecules which are synergistic or complementary. “A” and “B” are highly related with different pathways, and thus might affect to each other in unanticipated ways. For that reason, profiles of “A” and “B” are grouped as a set, then the ES (Enrichment Score) of “A and B” combination is calculated in two signaling pathways independently. So the similarity of expression pattern of “B” is now considered not only in “SP2” but also in “SP1” as a combinatorial drug partner. If “B” shows high ESs in both pathways, “B” could be a complementary partner for “A” as it covers “SP2” which “A” might not be able to regulate, and at the same time, synergistic effect could be expected in “SP1” as both of them are highly enriched in there.

Using these steps, the KS score was computed using these profiles. Then, random permutation tests (10,000 times) were carried out to estimate the significance of a distribution of those profiles. The molecules with p-value <0.01 were assumed as significant.

### Drug Ranking

At this point, we have listed single/combinatorial drugs for each disease-associated signaling pathway in our database. The goal of creating this system is to provide a means of selecting single/combinatorial drugs that can regulate disease-related signaling pathways to the greatest potential. To this end, for each drug, the number of pathways scored greater than the positive threshold was counted. The positive threshold for single drug and combinatorial drug were 0 and 0.5, respectively. The drugs were ranked in descending order of the number of pathways they appeared in. Pathways that scored less than the negative threshold were also listed. The negative threshold for single drug and combinatorial drug were 0 and −0.5, respectively. These negatively correlated pathways can be treated as negative effects.

### Cell Culture and Materials

A549 and MDA-MB-231 were purchased from American Type Culture Collection. RPMI containing 10% fetal bovine serum and 1% antibiotics were used for cell cultivation. Alsterpaullone, Scriptaid, Irinotecan hydrochloride, Semustine, Halofantrine hydrochloride, Vinblastine sulfate salt were purchased from Sigma.

### MTT Assay

A549 or MDA-MB-231 cells were seeded in the 96-well plates. After 24 h, cells were treated with indicated chemicals. After incubation for 3 days, MTT reagent (5 mg/ml) (Sigma) was added to each well, and the plate was placed at 37°C for 2 h. After aspirating the supernatant, 200 µl of dimethyl sulfoxide (Sigma) was added to each well. Colored formazan product was assayed spectrophotometrically at 570 nm using ELISA plate reader.

### Combination Index (CI) and Dose Reduction Index (DRI) Calculations

Synergism and antagonism for combinatorial drug were quantified by the combination index (CI), where CI<1, CI = 0, CI>0 indicate synergism, addictive, and antagonism, respectively. CI was determined by the following equation:
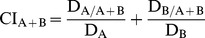



D_A_ is the concentration of drug A that induce the inhibition of cell growth. D_A/A+B_ is the concentration of drug A in the combination A+B giving the same inhibition effect. The dose reduction index (DRI) is a measure of how much the dose of each drug may be reduced in a combination for a given degree of effect compared with the concentration of each drug alone.
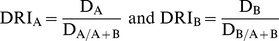



CI and DRI indexes were calculated with the CalcuSyn version 2.1 software (Biosoft, Cambridge, UK).

## Supporting Information

Information S1 **A case study on acute lymphoblastic leukemia (ALL) cells.** See the ranking of rapamycin in glucocorticoid resistance ALL cells. It proved that CDA does not heavily depend on the way of the signature extraction.
(DOC)
