# Supplementary material for: CDA: Combinatorial Drug Discovery Using Transcriptional Response Modules
Source: PLoS One. 2012 Aug 8;7(8):e42573. doi: 10.1371/journal.pone.0042573 (PMC3414439; doi:10.1371/journal.pone.0042573)
Supplement: Information S1 — A case study on acute lymphoblastic leukemia (ALL) cells. See the ranking of rapamycin in glucocorticoid resistance ALL cells. It proved that CDA does not heavily depend on the way of the signature extraction. (DOC) [file pone.0042573.s001.doc]

**Case study: Rapamycin for GC-resistance in acute lymphoblastic leukemia (ALL) cells**

Wei et al. demonstrated that rapamycin could reverse the glucocorticoid resistance state to sensitive state . To compare the performances of CMap and CDA, we extracted gene expression signatures of glucocordicoid (GC) sensitivity/resistance in acute lymphoblastic leukemia (ALL) cells (thirteen sensitive and sixteen resistance, GDS2493) using two different methods with different p values. As shown in Table S1, CMap is highly dependent on extraction method. With signatures using signal-to-noise statistics which is the method Wei et al. used, Rapamycin ranked on second position. However, the ranking fell to 146th and 307th with signatures from Limma, with p value < 0.01 and p value < 0.05, respectively. On the other hand, CDA shows more stable performance. Rapamycin was ranked in top 10 with any of signatures in CDA. This is because unlike CMap consider gene signatures as a set, CDA selectively choose genes participate on signaling pathways, and treat them as signaling pathway gene sets.

Although genomewide expression analysis with DNA microarray has become a routine tool in genomic research, extracting biologically meaningful information remains a major challenge. Statistically significant genes can be obtained by number of different ways. And there is no standard rule to restrict the number of genes. Significant gene selection is quite depending on individual researchers. As there are multiple ways, significant gene lists are diverse according to extraction algorithms and research principles. This diversity has the risk of insufficient information usage and could lead to inaccurate final interpretation. So we hypothesized that it is more appropriate to use functionally important genes rather than entire statistically selected genes for expression analysis and interpretation. And this hypothesis was validated by this rapamycin case.

**Table S1. Ranking of rapamycin in GC-resistant ALL cells**

| **DEG extraction method** | **Num of signatures** | **Rank in CMap** | **Rank in CDA** |
| --- | --- | --- | --- |
| Signal-to-noise  (p <= 0.0005) | 157 | 2 | Not found |
| Signal-to-noise  (p <= 0.001) | 244 | 2 | 10 |
| Limma  (p <= 0.01) | 391 | 146 | 5 |
| Limma  (p <= 0.05) | 543 | 307 | 6 |

**References**

1. Wei G, Twomey D, Lamb J, Schlis K, Agarwal J, Stam RW, Opferman JT, Sallan SE, den Boer ML, Pieters R *et al*: **Gene expression-based chemical genomics identifies rapamycin as a modulator of MCL1 and glucocorticoid resistance**. *Cancer cell* 2006, **10**(4):331-342.
